# Unraveling gene expression profiles in peripheral motor nerve from amyotrophic lateral sclerosis patients: insights into pathogenesis

**DOI:** 10.1038/srep39297

**Published:** 2016-12-16

**Authors:** Nilo Riva, Ferdinando Clarelli, Teuta Domi, Federica Cerri, Francesca Gallia, Amelia Trimarco, Paola Brambilla, Christian Lunetta, Alberto Lazzerini, Giuseppe Lauria, Carla Taveggia, Sandro Iannaccone, Eduardo Nobile-Orazio, Giancarlo Comi, Maurizio D’Antonio, Filippo Martinelli-Boneschi, Angelo Quattrini

**Affiliations:** 1 Experimental Neuropathology Unit, Institute of Experimental Neurology, Division of Neuroscience, San Raffaele Scientific Institute, Milan, Italy; 2 Laboratory of Genetics of Complex Neurological Disorders, Institute of Experimental Neurology, Division of Neuroscience, San Raffaele Scientific Institute, Milan, Italy; 3 2Neurology, IRCCS Istituto Clinico Humanitas, Milano University, Milan, Italy; 4 Axo-glia interactions Unit, Institute of Experimental Neurology, Division of Neuroscience, San Raffaele Scientific Institute, Milan, Italy; 5 NEuroMuscular Omnicentre (NEMO), Niguarda Ca Granda Hospital, Milan, Italy; 6 Hand Surgery and Microsurgery Unit, IRCCS Humanitas Clinical Institute, Milan, Italy; 7 3rd Neurology Unit, IRCCS Foundation “Carlo Besta” Neurological Institute, Milan, Italy; 8 Department of Clinical Neurosciences, San Raffaele Scientific Institute, Milan, Italy; 9 Universita` Vita e Salute San Raffaele, Milan, Italy; 10 Division of Genetics and Cell Biology, San Raffaele Scientific Institute, Milan, Italy

## Abstract

The aim of the present study is to investigate the molecular pathways underlying amyotrophic lateral sclerosis (ALS) pathogenesis within the peripheral nervous system. We analyzed gene expression changes in human motor nerve diagnostic biopsies obtained from eight ALS patients and seven patients affected by motor neuropathy as controls. An integrated transcriptomics and system biology approach was employed. We identified alterations in the expression of 815 genes, with 529 up-regulated and 286 down-regulated in ALS patients. Up-regulated genes clustered around biological process involving RNA processing and protein metabolisms. We observed a significant enrichment of up-regulated small nucleolar RNA transcripts (p = 2.68*10-11) and genes related to endoplasmic reticulum unfolded protein response and chaperone activity. We found a significant down-regulation in ALS of genes related to the glutamate metabolism. Interestingly, a network analysis highlighted *HDAC2,* belonging to the histone deacetylase family, as the most interacting node. While so far gene expression studies in human ALS have been performed in postmortem tissues, here specimens were obtained from biopsy at an early phase of the disease, making these results new in the field of ALS research and therefore appealing for gene discovery studies.

Amyotrophic lateral sclerosis (ALS) is the most common and severe form within the group of motor neuron diseases (MND), characterized by degeneration of both upper and lower motor neurons, leading to death two to five years after diagnosis^1^,^2^. While most ALS cases apparently occur sporadically (sALS), up to 10% have an affected relative and are considered familial (fALS) cases. In spite of recent advances in unraveling the genetic etiology of ALS^3^, mechanism/s of disease initiation and progression still remain elusive, even if protein misfolding and aggregation, dysregulation of RNA processing, proteasome impairment, neuro-inflammation, excitotoxicity, cytoskeleton and mitochondrial dysfunctions have been proposed^4^,^5^. It has been recently demonstrated that motor neuron cell death in ALS is profoundly influenced by neighboring non-neuronal cells, including glial cells, thus implicating a non-cell autonomous mechanism in disease pathogenesis^5^,^6^. Neurons are extremely polarized cells and neuronal cell body size is exceeded by several orders of magnitude by the length of axons, which represent up to 99% of motor neuron volume. The loss of peripheral motor nerve fibers is a major determinant of disability in patients with ALS and the peripheral nervous system (PNS) is involved at an early stage in the pathogenetic cascade, before symptom onset. In spite of this, possible detrimental factors within or to the PNS have not been extensively examined so far. Therefore, studying the PNS, including cells in contact with the full length of peripheral axons, such as Schwann cells, endothelial, interstitial and inflammatory cells, and their environment, could constitute an important, alternative approach in order to define the non-cell autonomous mechanisms in ALS^5^.

Most specimens from ALS patients are obtained post-mortem, de facto limiting the possibility of performing studies revealing pathogenetic mechanisms underlying the earliest stages of disease. In clinical practice, early differential diagnosis between MND and motor neuropathies (MN) is extremely important, as prognosis and therapeutic approach are different. However, there are no reliable biomarkers for ALS diagnosis or disease progression rate. Over the last years, we validated the motor nerve biopsy as a reliable technique for MND diagnosis from the earliest stages of disease^7^. Taking advantage of this unique repository of human nerve biopsies we investigated the molecular pathways underlying ALS pathogenesis within the PNS, using a transcriptomic and system biology approach. Understanding early events in pathogenesis could be highly valuable for diagnosis, treatment and development of novel potential biomarkers.

## Results

### Motor nerve biopsy from ALS and motor neuropathy patients

**Figure 1:**
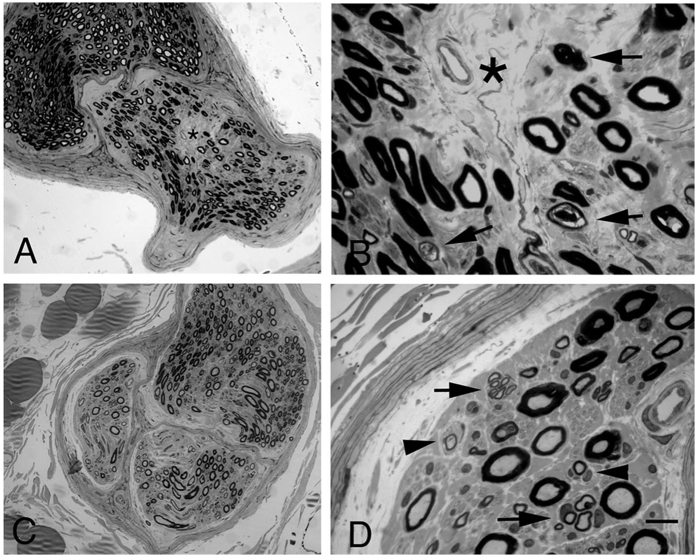
Representative neuropathological cases. Transverse semi-thin sections of biopsy of motor nerve from an ALS case (**A** and **B**) and motor neuropathy patient (**C** and **D**). In ALS patients, focal decreased density of myelinated nerve fibers (**A** and **B**: asterisk) is evident in a nerve fascicles. At higher magnification (**B**), axonal degeneration is evident (arrows). (**C**) Diffuse mild reduction of myelin nerve fibers is present in a representative section from patients with definite diagnosis of motor neuropahty. There are clusters of small myelinated fibers (**D**, arrows), indicating axonal regeneration. In addition small onion bulbs (**D**, arrowheads), indicating remyelination, are present. Bar: (**A**,**C**) 50 μm; (**B**,**D**) 10 μm.

Patient’s clinical, pathological and demographic information are provided in Supplementary Table S1. Mean age at biopsy and sex distribution did not differ between groups (mean age: ALS: 57.6 years, SD: 11.2; MN: 57.7 years, SD: 17.9; Females to Males ratio: ALS: 1/7; MN. 4/3), while, as expected, disease duration was significantly longer in MN patients compared with ALS patients (mean disease duration: ALS: 16.7 months, SD: 10.0; MN: 64.2 months, SD: 48.8; p < 0.05). The mean ALS- Functional Rating Scale-revised value was 38.5 (SD: 4.3), while for MN patients the mean Overall Disability Sum Score was 2.7 (SD: 1.8). The diagnostic neuropathological features of ALS and MN patients are presented in Fig. 1. As reported elsewhere, the morphological aspects of the motor nerves have been shown to differ in patients with a definitive diagnosis of MND or MN^7^. Signs of active axonal degeneration, focal fibers loss and low axonal regeneration suggest a pathological diagnosis of MND. Instead, signs of demyelination/remyelination and a high number of regenerating clusters, indicating axonal regeneration, support a pathological diagnosis of MN.

All patients underwent motor nerve biopsy before the institution of specific therapies (Supplementary Table S1).

### Differential Expression Analysis in ALS patients

Gene expression analysis was performed on motor nerves from ALS and MN patients to identify alterations in gene transcription associated with the disease. Differential expression was assessed on the 27,679 probes that passed the filter criterion (58% of total, see Materials and Methods). A Principal Component Analysis (PCA) score plot for the first 3 PCs and a bar plot with percentage of explained variance is reported in Supplementary Figure S1, which shows a separation between ALS and MN samples along PC1.

**Figure 2:**
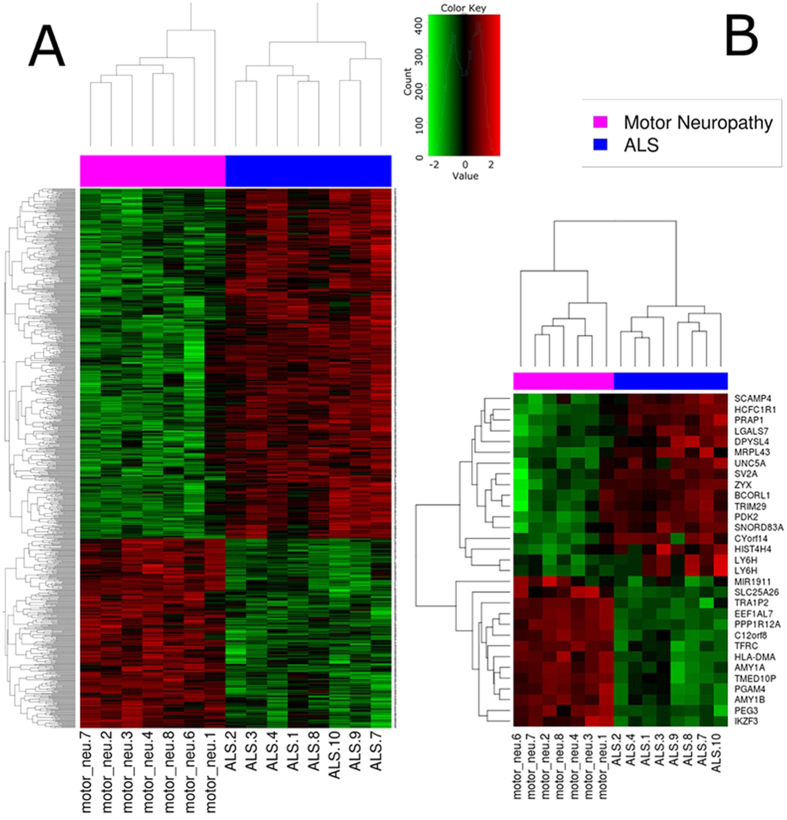
Heatmap representing hierarchical clustering of the 15 samples (columns) and genes (rows) detected as differentially expressed according to two different thresholds. (**A**) FDR < 0.01 and FC > 1.5 or FC < 0.66; (**B**) FDR < 0.05 and FC > 3 or FC < 0.33. The expression level of each gene has been standardized by subtracting the gene’s mean expression level and dividing by the standard deviation across all samples. This scaled expression value is plotted in red-green scale color, with red indicating higher expression and green lower expression in ALS patients.

Limma method identified a total of 815 DEGs: of these, 529 were up-regulated and 286 down-regulated in ALS patients. A heatmap was generated, showing a clear pattern of bi-clustering of up- and down-regulated probes across the two compared conditions (Fig. 2). A list of the most strongly up- and down-regulated DEGs (False Discovery Rate: FDR < 0.05 and FC > 2.8 or FC < 1/3) in ALS patients is reported in Tables 1 and 2. Proline-rich acidic protein 1 (*PRAP1*) was the top up-regulated gene (FC = 4.57, p-adjusted = 9.05*10-4). *PRAP1* is expressed in epithelial cells and plays a role in cell-cycle control, promoting cell-survival; however, its role in the central nervous system and PNS is unknown^8^. The most down-regulated gene was a member of the family of the heat shock protein 90 kDa beta (member 3, pseudogene): *HSP90B3P* (FC = 0.032, p-adjusted = 8.60*10-4) with unknown molecular function up to date^9^.

We observed in ALS patients a significant enrichment of small nucleolar RNA (snoRNAs) transcripts: 31 snoRNAs, against an expected number of 7.9 (ratio = 3.89, p = 2.68*10-11; hypergeometric test) were detected in the list of 815 DEGs among upregulated genes (Table 3).

### DEG validation by qRT-PCR and Immunohistochemistry

For a subset of transcripts, quantitative real-time RT-PCR and immunohistochemistry were used to corroborate and validate microarray results. Genes were selected based on fold changes (>2) and functions such as genes involved in the development of the nervous system (*LMNA* and *TFRC*), axon guidance (*DPYSL4*), RNA processing and small ribosomal subunit assembly (*NOP14*) and mitochondrial functions (*MRLP43*). The results of quantitative RT-PCR validation, performed on three randomly selected samples for each group, demonstrated a positive correlation with the microarray data for four of the selected genes, while for MRLP43 the correlation was not significant (Supplementary Tables S2 and S3; Supplementary Figures S2 and S3).

**Figure 3:**
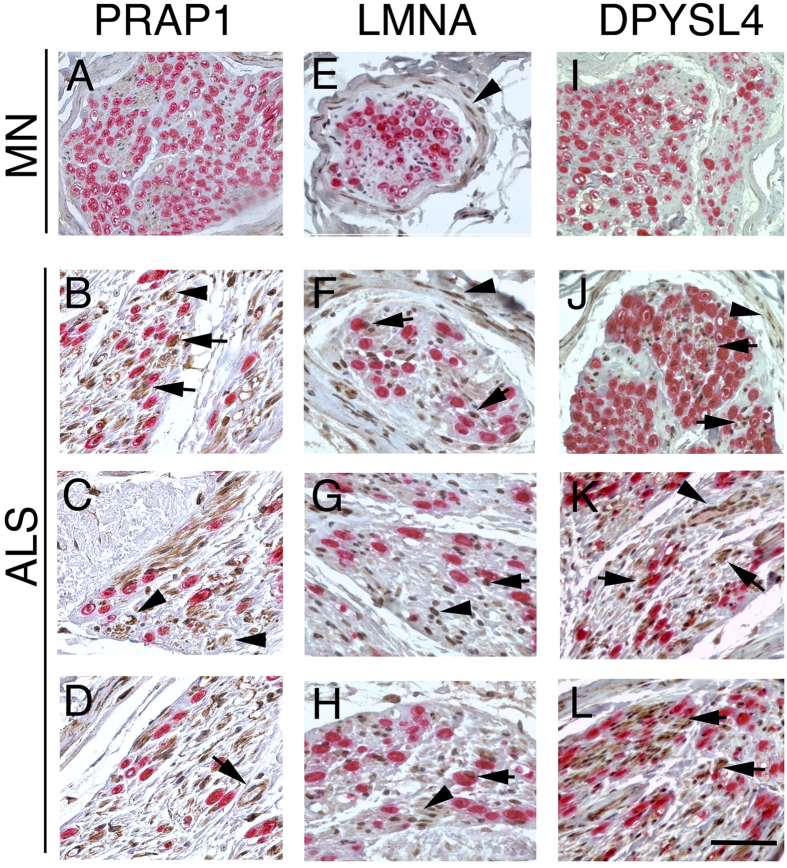
Immunohistochemistry localization of PRAP1, LMNA and DPYSL4 in MN and ALS motor nerves. Double staining for MBP (red), to mark myelinated nerve fibers, and PRAP1 (**A**–**D**), LMNA (**E**–**H**) and DPYSL4 (**I**–**L**) (brown) shows increase expression in ALS motor nerves. PRAP1 immunoreactivity was present in the endoneurium in numeorus cells (arrowheads, **B**,**C**) including cytoplasm of Schwann cells (arrows, **B**; MBP immunoreactivity identified the myelin sheath) and endothelial cells (arrow, **D**). LMNA shows immunoreactivity in the perineurium in both MN and ALS nerves (arrowhead, **E**,**F**); in ALS motor nerves, immunoreactivity was present in nuclei and cytoplasm of all endoneurial cells (arrowhead, **G**,**H**) and Schwann cells (arrows, **F–H**). In ALS motor nerves, DPYSL4 was expressed in perineurium (arrowhead, **J**) and in the cytoplams and nuclei of many cells (arrows in **J**–**L**) including endothelial cells (arrowhead, **K**) and Schwann cells (arrows **J**,**K**). Bar: (**A**,**E**,**I**) 50 μm; (**F–H**,**J–L**) 50 μm.

**Figure 4:**
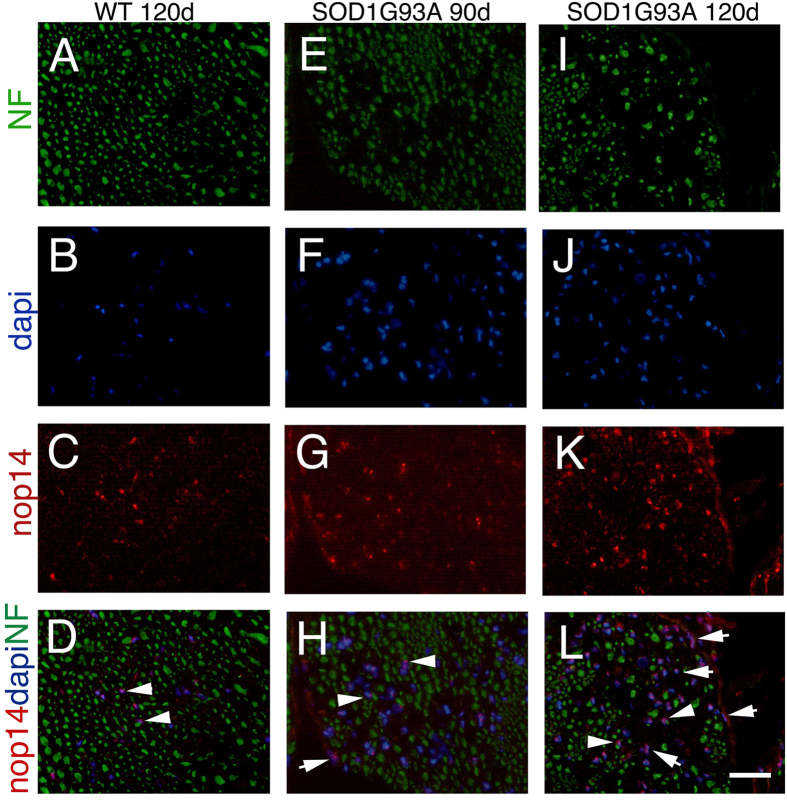
Expression of NOP14 in the sciatic nerve of normal and SOD1^G93A^ mice. Tranverse sections from wild-type (**A**–**D**), SOD1^G93A^ mice at 90 days (diseases onset) (**E**–**H**) and 120 (end-stage disease) (**I**–**L**) double-stained for neurofilament (green), to recognize axons, and NOP14 (red). DAPI staining of nuclei (blue). Nop14 immunoreactivity was present in cell nuclei in wild-type nerves (arrowhead, merge in **D**); NOP14 shows an increase expression in SOD1^G93A^ nerves in cell nuclei of the Schwann cells (arrowhead, merge in **H** and **L**); perineurial cells were also positive for NOP14 (merge in **H** and **L**, arrows). Bar: (**A–L**) 120 μm.

To establish the localization and the pattern of expression for PRAP1, LMNA, DPYSL4 and NOP14 in the PNS, we performed immunohistochemistry analysis on cross sections of human motor nerves and SOD1^G93A^ mice sciatic nerves. We showed a significant correlation between mRNA and protein levels for all selected proteins (Figs 3 and 4). PRAP1, LMNA and DPYSL4 displayed elevated signal in the ALS motor nerve (Fig. 3B–D,F–H and J–L) compared to the nerve from MN patients (Fig. 3A,E and I). In the current study, expression of PRAP1 immunoractivity was observed at high level ubiquitously throughout the endoneurium in motor nerves from ALS compared to MN nerves. PRAP1 was expressed in the cytoplasm of myelin-forming Schwann cells, identified by myelin basic protein (MBP), fibroblasts and endothelial cells (Fig. 3B–D). Conversely, both the nuclei and cytoplasm of the Schwann cells, other endoneurial and perineurial cells were labed by the LMNA antibody (Fig. 3F–H). Similarly to LMNA, in ALS nerves, we observed differences in the expression of DPYSL4 compared with MN nerves. DPYSL4 was expressed at high level in the endoneurium and perineurium, both in the nuclei and cytoplasm of the Schwann cells, endothelial cells and perineurial cells (Fig. 3J–L). We further investigated the expression of NOP14 in the SOD1^G93A^ mice model and control animals. Results showed similar expression of NOP14 in the nuclei of the Schwann cells, in both control nerves (Fig. 4A–D) and SOD1^G93A^ at 90 days (disease onset) (Fig. 4E–H). SOD1^G93A^ nerves showed increase expression of NOP14 in cell nuclei of the Schwann cells at 120 days (end-stage disease) (Fig. 4I–L), correlating with disease progression. To the best of our knowledge, this is the first demonstration of PRAP1, LMNA, DPYSL4 and NOP14 expression and localization in ALS nerves.

To confirm further the Schwann cells origin of the transcripts, we compared our data on DEGs with a gene profiling study on normal and neuropathic mouse nerves^10^ (see Material and Methods); this analysis suggested that most up- or down-regulated genes are expressed in peripheral nerve and thus are likely Schwann cell specific (Tables 1 and 2).

### Pathway Analysis

DEGs were then classified into functional groups according to Gene Ontology (GO) annotation tools. Functional enrichment analysis revealed 16 and one GO terms significantly enriched with DEGs in up- and down-regulated genes, respectively. As shown in Table 4A, terms enriched with genes up-regulated in ALS patients were ‘response to unfolded protein’ (FDR = 0.008) along with its descendant term ‘endoplasmic reticulum unfolded protein response’ (FDR = 0.008) and ‘positive regulation of nuclease activity’ (FDR = 0.03). Genes down-regulated in ALS were over-represented in biological processes involving glutamate metabolism: ‘transferase activity, transferring nitrogenous groups’ (FDR = 0.01). These include *CCBL2*, also called *KAT3* (Kinurenine Aminotransferase III), which is involved in the synthesis of kynurenic acid (KYNA), an important endogenous antagonist of NMDA and alpha 7-nicotinic acetylcholine receptors^11^.

We further tested over-representation of DEGs in the context of Reactome database^12^. This analysis revealed enrichment of prominent changes in up-regulated genes in four pathways (Table 4B). We consistently retrieved genes related to ‘Metabolism of proteins’ (FDR = 0.0082) and the three hierarchically related pathways: ‘Unfolded Protein Response (UPR)’ (FDR = 0.0028), ‘Activation of Chaperones by IRE1alpha’ (FDR = 0.0072) and ‘Activation of Chaperone Genes by XBP1(S)’ (FDR = 0.0072). The IRE1alpha-XBP1 pathway plays relevant roles in both physiological and pathological conditions, modulating target genes involved in lipid synthesis, endoplasmatic reticulum (ER) protein degradation and protein folding^13^. Notably, among the up-regulated genes annotated in these pathways Lamin A/C (*LMNA*) is required for normal development of skeletal muscle and PNS; moreover, mutations in the *LMNA* gene are associated with muscular dystrophy and hereditary peripheral neuropathy (Charcot-Marie-Tooth disease)^14^,^15^.

### Network Analysis

**Figure 5:**
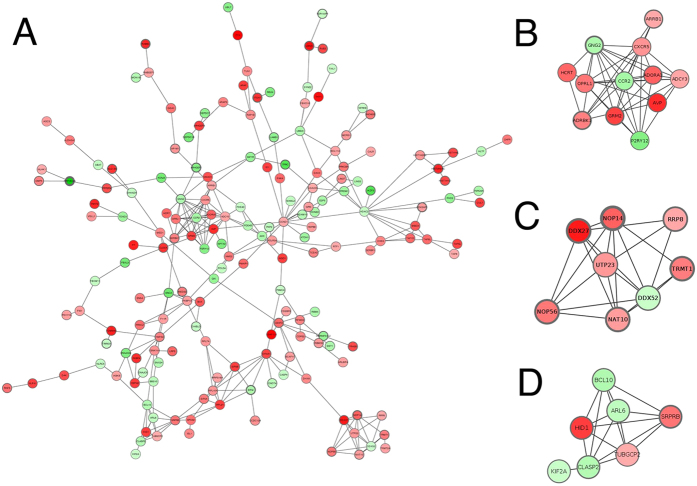
Interaction networks among differentially expressed genes as retrieved from STRING database at confidence score >0.7. (**A**) Largest connected network, with subnetwork modules mined by ClusterONE algorithm as densely connected regions, highlighted in (**B**–**D)**. Nodes are color-coded according to values of log2 (fold-change), with red and green nodes representing up-regulated and down-regulated genes respectively, and color saturation prorportional to level of modulation. Node size is proportional to degree connectivity and border thickness is proportional to adjusted p-value of association.

Of the 815 DEGs, 766 were mapped on STRING human interactome database for constructing interaction network^16^. Of these, 266 nodes were interacting with 343 edges distributed over 35 connected components, which were globally enriched in interactions (observed 343; expected: 274; p = 3.5*10-5 according to STRING null random network model). *HDAC2*, belonging to the histone deacetylase family, was the most interacting node (Fig. 5A). *HDAC2* plays an important role in transcriptional regulation and modulates relevant neuronal processes such as neuronal differentiation^17^; furthermore, HDAC1 and HDAC2 are essential for Schwann cells survival and myelination by the induction of transcriptional factors for myelin genes^18^,^19^.

Cluster analysis on the largest connected component (181 nodes, 287 interactions) highlighted three modules (Fig. 5B–D): sub-network-1 (n = 12, Q = 0.72, p = 5.7*10-5), sub-network-2 (n = 8, Q = 0.91, p = 3.0*10-4) and sub-network-3 (n = 7, Q = 0.72, p = 2*10-3). GO enrichment analysis was performed on member genes of these sub-networks. The most specific over-represented terms for the three modules are shown in Table 5: sub-network-1 is enriched with genes related to G-protein coupled receptor signalling pathway (FDR = 5.11*10-5); ribosomal RNA processing is over-represented in genes belonging to sub-network-2 (FDR = 2.78*10-7) and the third module was characterized mainly by genes involved in microtubule cytoskeleton organization (FDR = 0.0052).

Regarding active module search, the two modules with the highest score (S1 = 16.13, S2 = 6.8) were selected and are displayed in Supplementary Figures S4A and S5A. Module-1 is composed of 338 genes and 1378 edges, whereas module-2 involves 189 nodes interacting with 833 edges. Pathway analysis revealed that module-1, a sub-network centered on *UBC* hub gene (degree = 270), was over-represented of genes participating to, among others, “protein modification by small protein conjugation” (FDR = 1.05*10-5), “mRNA processing” (FDR = 2.4*10-6) and “RNA splicing” (FDR = 5.8*10-6). As regarding module-2 (Supplementary Figure S5A), we found that “histone modification” was the most significantly over-represented Biological Process GO term among the most specific terms (FDR = 9.5*10-13). This module was enriched of genes involved in “histone methyltransferase activity” (FDR = 8.8*10-5) and in “NAD-dependent histone deacetylase activity” (FDR = 3.6*10-9): indeed, again, *HDAC2* was the top-interacting node (degree = 50), also exhibiting the higher clustering coefficient (c = 0.42) which is a measure of cohesiveness of the node’s first-order neighbors, revealing the important role played by this down-regulated gene. Other enriched specific gene-sets were the “negative regulation of transcription from RNA polymerase II promoter” (FDR = 9.5*10-9), and the “delayed rectifier potassium channel activity” (FDR = 1.1*10-7). The most over-represented specific GO terms are reported in Supplementary Figures S4B and S5B.

## Discussion

This study was based on previous evidence suggesting that the early events in ALS pathogenesis may be influenced by the PNS cells, such as Schwann cells, and environment, exerting a non-cell autonomous effect on motor neuron axons^5^,^6^. Our aim was to identify genes, molecular pathways and biomarkers relevant for human ALS. The results of our study show, for the first time, specific gene expression changes in motor nerve biopsies of sporadic ALS patients as compared with MN patients. We identified both pathways previously reported to be relevant for ALS, supporting potential pathogenetic causality^4^,^5^, as well as novel potential players in ALS pathogenesis.

Pathways significantly enriched in up-regulated genes include metabolism of protein, UPR, ER stress, activation of chaperones and positive regulation of nuclease activity. Coherently, GO analysis performed on active subnetwork module-1 showed a significant over-representation of pathways related to ubiquitin-protein ligase activity, SUMO ligase activity and Ubiquitin C was the most relevant hub gene.

Impaired protein degradation is a well-recognized potential causative effect in ALS pathogenesis, supported by the discovery of fALS-linked mutations affecting proteins that are directly involved in proteostasis such as SOD1, which account for about 20% of fALS, but also ubiquitin 2 (UBQLN2) and Optineurin (OPTN)^3^. Mutant SOD1 misfolds, accumulates as oligomers in the ER and later as aggregates, leading in turn to activation of UPR^20^. Indeed the UPR aim is to restore proper folding by stalling protein translation; however, when unsuccessful, it determines apoptosis^21^. Accordingly, in ALS patients we found up-regulation of genes related to activation of chaperones by the IRE1/XBP1 pathway, one of the most important stress sensors controlling the UPR^13^. Interestingly, the UPR can be modulated by guanabez, a drug approved for the treatment of hypertension, which shown a potential neuroprotective effect in the SOD1^G93A^ mouse model of ALS^22^.

No previous study has studied gene expression profiles in motor nerves of ALS patients at disease onset, therefore, no direct comparison is possible. However, a recent systematic meta-analysis has highlighted genes that were found to be differentially expressed at least two times: between the DEGs identified, six are also shared with our study, namely calcium binding protein 1 (*CABP1*), cathepsin B (*CTSB*), granulin (*GRN*), calreticulin (*CALR*), Rh-Associated Glycoprotein (*RHAG*) and proteolipid Protein 1 (*PLP1*)^23^. Of these, *CTSB* and *GRN* were replicated in more than one mouse study and in at least one human study^24^,^25^,^26^,^27^. *CTSB* belong to a family of lysosomal proteases^26^,^27^ while *GRN*, similarly, is another gene involved in protein turnover whose mutations are associated with frontotemporal lobar degeneration with ubiquitin-positive inclusions^28^.

Interestingly, CALR is not only a key protein in the maintenance of Ca2+ levels inside the ER, but is also a chaperone protein, regulating the folding of newly synthesized proteins^29^. Moreover, we found five DEGs overlapping between our study and a gene expression study, even though it was performed in late-stage human ALS post-mortem spinal cord: *PLP1,* Protein kinase C zeta (*PRKCZ*), Cathepsin B (*CTSB*), Ankyrin G (*ANK3*) and Islet-1 (*ISL1*)^26^. Notably, *PLP1* encodes for the major myelin proteolipid protein, that plays a role not only for stabilization and maintenance of myelin sheaths, but also for axonal survival; mutations in this gene cause spastic paraplegia type 2 and Pelizaeus-Merzbacher disease^30^. Interestingly, ANKG is a key component of the nodes of Ranvier and mutations in *ANK3* are associated with psychiatric disorders including schizophrenia, autism and bipolar disorder^31^.

In ALS motor nerve, we also observed the differential expression of many genes and pathways related to RNA processing. ALS patients showed increased expression of snoRNAs, a large family of small non-coding RNAs (ncRNAs) which represent a class of regulatory RNAs responsible for post-transcriptional maturation of ribosomal RNAs (rRNAs). SnoRNAs guide modification of rRNA and snRNA nucleotides, influence alternative splicing of complementary pre-mRNAs, and control translation and stability of mRNAs through RISC-dependent activity. Thus, we cannot exclude a direct role of snoRNAs in ALS pathogenesis, considering the growing evidence for a role of snoRNA and other ncRNAs in neurological diseases^32^. Moreover, recent studies have independently demonstrated several non-canonical functions for snoRNAs, including miRNA-like capabilities. In turn, miRNAs dysregulation may play a critical role in neurological disorders pathogenesis, including ALS^33^. In agreement, two genes with a recognized role in RNA metabolism and miRNAs biogenesis, namely TAR DNA-binding protein-43 (*TDP43*) and fused in sarcoma/translocated in liposarcoma (*FUS/TLS*), are mutated in fALS cases^34^. Finally, four hub genes in network analysis were related to ribosomes (*RPL10A* and *RPL21*) and RNA metabolism (*EIF4A1* and *U2AF2*) and in identified sub-networks we observed a significant enrichment of ncRNA processing and ribosome biogenesis, confirming the relevance of these biological processes in ALS. Moreover, in keeping with these results, GO analysis performed on active subnetwork module-1 showed a significant over representation of pathways related to mRNA processing and splicing.

Pathway analysis showed in ALS patients major down-regulations in genes related to glutamate metabolism, such as *CCBL2*, also called *KAT3* (Kynurenine aminotransferase), supporting the role of glutamate-induced excitotoxicity in ALS pathogenesis, coherently with previous reports^35^. KAT3 catalyzes the synthesis of kynurenic acid (KYNA), a known endogenous antagonist of the NMDA (n-methil-D-aspartate) glutamate receptors. KYNA is believed to play a role in modulating glutamate-mediated neurotransmission^11^. Abnormal concentrations of KYNA in brain tissue has been linked to a number of human neurodegenerative diseases^12^,^36^. The kynurenine pathway has recently emerged as a potential contributing factor in ALS pathogensesis, and could offer novel therapeutic target option for ALS^36^.

Neuro-inflammation and dysregulated expression of cytoskeleton related genes have been implicated in ALS^37^,^38^. In ALS motor nerves, our analysis did not reveal any significant over-representation of inflammatory pathways, indirectly supporting the hypothesis that inflammation could be secondary to nerve fibers damage and hence induced in both diseases^39^. However, *CCR2* was down-regulated in ALS. CCR2 and its ligand monocyte chemoattractant protein-1 (MCP-1) play a role in the macrophage inflammatory response following PNS injury, and promote nerve regeneration^40^. CCR2 and MCP-1 have previously been reported to be implicated in both ALS^41^ and peripheral neuropathies pathogenetic cascade^42^. Our finding corroborates the hypothesis that defective monocyte/macrophages infiltration at the site of nerve degeneration may be implicated in ALS. Instead, the network analysis recognized genes belonging to the microtubule cytoskeleton organization, suggesting impairment in axonal transport, in agreement with a previous study analyzing the sciatic nerve of the SOD1^G93A^ transgenic mice^38^. Nonetheless, we cannot exclude the possibility that cytoskeleton-related genes dysregulation might reflect a response to injury process rather than having a specific role in disease pathogenesis and neurodegenerative triggering.

Notably, network analysis of DEGs highlighted *HDAC2*, belonging to the histone deacetylases family, as the most interacting node, which was coherently also the top-interacting node of active subnetwork module-2, showing a significant over-representation of pathways related to histone-deacetilase, histone methyltransferase activities, the nucleosome remodeling and deacetylase complex (NuRD). HDAC2 plays an important role in transcriptional regulation and modulates relevant neuronal processes such as neural differentiation and axonal regeneration^17^, opening new potential windows of pharmacological interventions in neurodegenerative disease. Furthermore, HDAC1 and HDAC2 are essential for Schwann cells survival and myelination due to the induction of transcriptional factors for myelin genes^18^,^19^. In the context of ALS, transcriptional dysregulation is recently an emerging factor in the progression of the disease^43^. It has been proposed that HDAC6 regulates aggresome formation of misfolded proteins in mutant SOD1^44^. Furthermore, the inhibition of HDACs with trichostatin A reduces axonal degeneration and increases lifespan of ALS mice^45^, although it remains almost unknown the contribution of HDACs to motor neuron survival and functioning in ALS.

Previous studies have shown that neighboring glial cells exerting a non-cell autonomous effect on motor neurons have a crucial role in ALS pathogenesis^5^,^6^. Although microglia and astrocytes have been recognized as important factors in ALS^5^, the role of other glial cells, such as Schwann cells, has not been thoroughly investigated so far. Recent studies show conflicting results on Schwann cells involvement in the ALS progression. Increase expression of SOD1^G93A^ exclusively in Schwann cells of the transgenic mice showed a beneficial effect on disease^46^. While elimination of mutant SOD1^G37R^ determined a worsening in disease progression, suggesting an unexpected neuroprotective role for the mutant SOD1 Schwann cells^47^. Nevertheless, excision of SOD1 in Schwann cells of SOD^G85R^ mice delayed disease onset and increase survival^48^. We acknowledge that we extracted the total RNA from motor nerve samples which is an heterogeneous tissue, containing the axons of motor neurons and their ensheathing Schwann cells, perineurial cells, fibroblasts and endothelial cells; therefore, it is not possible to state which cells within the nerve are expressing each individual DEG. However, selected DEGs were expressed presumably by Schwann cells as showed by immunohistochemistry and gene profiling studies on normal and neuropathic mouse nerves^10^, where more than 90% of the mRNA is of Schwann cell origin. Taken together, our data suggest that PNS environment and possibly Schwann cells might exert a role in the non-cell autonomous ALS motor neuron degeneration^49^, potentially pertaining either ALS causality or progression. Indeed, variants in two of our DEGS, a Zinc Finger Protein 512B (*ZNF512B*) and *PGN* have been previously reported as either prognostic factor or associated to susceptibility to ALS^50^,^51^,^52^.

Our study is unique as it investigates PNS and its relevance for human ALS by taking advantage of human diagnostic motor nerve biopsy samples from early stage of the disease. It is a common dilemma for gene expression studies the inference of causality: transcriptomic changes may be a consequence of ALS, rather than its cause^53^. Nonetheless, many of the pathways highlighted by our experiments turned out to be involved in ALS, supporting potential pathogenetic causality. Moreover, while we acknowledge that one potential limitation of our study may be the lack of normal control motor nerves, by comparing two groups of patients with similar diseases involving the PNS may also have limited the possibility of highlighting conserved, unspecific response-to-injury process. Remarkably, genes chosen for immunohistochemistry validation studies gave results consistent with the microarray data showing for the first time the cellular expression of our selected DEGs in motor nerves. To our knowledge, this is the first study to identify *in vivo* the expression and cellular localization in the human motor nerve of PRAP1, DPYSL4 and LMNA associated with ALS. Further studies are required to determine the functional significance of these molecules in the progression of ALS.

In conclusion, we define early molecular pathways within the PNS relevant for human ALS. Since extensive damage in ALS already occurs by the time clinical symptoms appear, the ability to perform an early diagnosis and therapeutic intervention, ideally at the pre-symptomatic stage, could have a more beneficial neuroprotective effect on disease. We do not know if our results would be useful in a clinical setting, especially since motor nerve biopsies are rarely performed; however the relevance of candidate genes reported in the present study may be a source of clinical biomarkers for ALS.

## Materials and Methods

### Human motor nerves

We analyzed gene expression changes in human obturator motor nerve diagnostic biopsies obtained from eight sALS patients at disease onset and from seven patients affected by MN, which were used as controls. Normal control motor nerves were not available for this study because we followed the strict indication criteria for considering the motor nerve biopsy (Supplementary Table S1). Thus, we have decided to performed comparative studies using motor nerve biopsies from two groups of patients to discover transcriptomic changes that could account for the differences in their clinical course.

In ALS patient, following clinic-pathologic diagnosis, mutations in *TARDBP, FUS* and *SOD1* genes as well presence of GGGGCC hexanucleotide repeat expansion in the first intron of *C9Orf72* were excluded. The presence of the GGGGCC hexanucleotide expansion in the first intron of C9orf72 status was assessed using a repeat-primed PCR assay^54^ on an automated ABI3730 DNA-analyzer. A amplicon-length PCR was performed to define the length of normal alleles (up to 30 repeats) and to exclude a pathological repeat expansion when two normal alleles are detected. In addition, the presence of the GGGGCC expansion was confirmed on a second DNA extraction and applying a different repeat-primed PCR protocol^55^. A cut-off of ≥30 repeats combined with a typical sawtooth pattern was considered pathological. All the coding exons and 50 bp of the flanking intron-exon boundaries of *SOD1*, of *TARDBP* and of *FUS* have been PCR amplified and a direct sequence analysis was performed using the Big-Dye Terminator v1.1 sequencing kit (Applied Biosystems Inc.), and run on an ABIPrism 3730 genetic analyzer.

All human nerve biopsies had been obtained for diagnostic purposes, after informed consent, and stored in our tissue bank, all experimental protocol were approved by San Raffaele Scientific Institute Ethical Committee (Milan, Italy), and the methods were carried out in accordance with the approved guidelines. The diagnostic biopsy of the anterior branch of the obturator nerve was performed under local anesthesia plus short sedation, as described. Briefly, each nerve was divided into three segments. One segment was fixed in 10% formaline for routine histology using paraffin embedded tissue. A second segment was fixed in 2% buffered gluteraldehyde and post-fixed in 1% osmium tetroxid. After alcohol dehydration, these samples were embedded in Epon. Transverse sections (0,5–1 μm) were stained with toluidine blue and examined by light microscopy. Ultrathin sections were stained with uranil-acetate and lead citrate and examined by electron microscope. The third nerve segment was frozen and stored in liquid nitrogen, and used for gene expression studies^7^.

We selected patients meeting the criteria for a pathological diagnosis of probable MND and probable or definite neuropathy, according to previously published criteria^7^. ALS clinical diagnosis was further confirmed at follow-up according to El Escorial diagnostic criteria^56^, while the diagnosis of neuropathy was confirmed according to standard criteria. Disability of MN patients was measured using the Overall Disability Sum Score^57^. For ALS patients, disease severity was assessed using the ALS Functional Rating Scale^56^.

### RNA extraction and gene expression analysis

Total RNA was isolated from motor nerve samples using the RNeasy kit (Qiagen, Venlo, Netherlands). RNA was quantified using the Nanodrop-2000 spectrophotometer (Celbio, Milan, Italy) and Agilent 2100 Bioanalyzer (Agilent Technologies, Palo Alto, CA) was used to assess RNA integrity. Gene expression study was performed using the IlluminaҐ HumanHT-12 v4 BeadChips (Illumina Inc., San Diego, California, USA), each individual array targeting more than 47,000 transcripts selected primarily from the NCBI RefSeq database (Release 38). We adopted the Illumina Whole-Genome Gene Expression DASLҐ HT Assay, which is optimized for degraded RNA^58^. An amount of 250 ng of total RNA was retrotranscribed to cDNA using biotinylated oligo (dT) and random nonamer primers. The biotinylated cDNA was then annealed to the DASL Assay Pool (DAP) probe groups, containing specific oligonucleotides of 50 bases designed to interrogate each target sequence in the transcripts. Then, universal PCR amplification and Cy3 staining steps were performed. Finally, the resulting labeled PCR products were hybridized to the BeadChips, which were imaged using the IlluminaҐ BeadArray Reader. The software IlluminaҐ GenomeStudio version 2011.1 was used to generate fluorescent hybridization signals.

### Pre-processing and differential expression

Quality controls and subsequent preprocessing steps were performed using R statistical environment^59^ and Bioconductor package lumi^60^. We assessed the presence of outliers by means of principal component analysis (PCA) and signal intensities distribution analysis with R package Array Quality Metrics functions^61^. Normalization of signals was performed with quantile procedure as available in lumi package. Subsequently, probes whose fluorescence signal was deemed close to estimated background were excluded: we retained probes that were called ‘present’ by GenomeStudio algorithm (detection call p-value < 0.05) on at least 30% of samples in at least one of the two groups^62^. We performed PCA on the normalized filtered set of 27,679 probes. The 15 samples were projected on the first 3 PCs, which overall explain more than 50% of the variance of expression.

Differential expression analysis on normalized and filtered dataset was performed using limma method^63^, which relies on a linear model and pooled estimate of gene variance to detect DEGs. Correction for multiple testing was done by controlling the False Discovery rate (FDR) with Benjamini-Hochberg procedure^64^. Threshold values to declare DEGs were set to FDR < 0.01 and fold-change (FC) > 1.5 or fold-change <0.66.

### Pathway analysis

Functional enrichment analysis of up- and down-regulated DEGs was performed on Gene Ontology and Reactome databases, using WebGestalt^65^ and Bioconductor R package ReactomePA^12^. Over-representation for both databases was evaluated via hypergeometric test with Benjamini-Hochberg multiple testing adjustment^64^, using the filtered set of genes as customized background set. For Gene Ontology, we considered for the analysis only terms with at least 3 annotated genes.

### Network analysis

The identified DEGs were superimposed on their corresponding proteins available on interactome database STRING v10 (Search Tool for the Retrieval of INteracting Genes)^16^ and a functional and protein-protein interaction network, based on experiments, co-expression, literature-mining and databases, was retrieved, retaining only high confidence interactions (score >0.7). The retrieved networks were exported, visualized and analyzed using Cytoscape 3.0, followed by an assessment of centrality metrics (degree and betweeness) to reveal possible key-players within the set of DEGs.

Cluster analysis according to network connectivity was carried out with ClusterONE plugin^66^ in order to detect densely connected sub-networks, which are more likely to be considered as functional modules. We retained only clusters with at least 6 members and a quality score Q > 0.7 as a measure of cohesiveness. To assess biological relevance of mined modules, over-representation of Gene Ontology terms in subnetworks genes was evaluated with WebGestalt tool.

We next performed an “active sub-networks” extraction from global human interactome, which we downloaded from STRING v10 database, filtering interactions with confidence score>0.7. The network was imported in Cytoscape, mapping the filtered genes onto it and discarding all nodes not present in our set of filtered genes. The final analyzed network consisted of 268,759 edges involving 13,714 genes-nodes. We used jActiveModules plugin^67^ to identify active modules, i.e. sub-networks enriched of significantly DEGs and hence reflecting how they are “active” under the examined contrast. For each sub-network, jActiveModules computes an aggregate score with a size-adjusted sum of z-scores and employs simulated annealing as a search strategy for top-scoring modules across the interactome. The overlap threshold between modules was set to 0.2 and the search depth to 1. To assess biological meaning, we carried out functional enrichment analysis with WebGestalt using filtered genes as background.

### Array data validation by Real-Time PCR (qRT-PCR) and Immunohistochemistry

In order to validate the microarray results, expression levels of selected DEGs was confirmed by quantitative RT-PCR performed on five independent cDNA samples (randomly selected from the pool of patients, three from each group, ALS and MN). Transcripts were selected for validation based on having a large change in expression level (>2 fold) the absence of transcript variants and functions. We selected genes involved in development of the nervous system (*LMNA* and *TFRC*), in axon guidance (*DPYSL4*), RNA processing and small ribosomal subunit assembly (*NOP14*) and mitochondrial functions (*MRLP43*). GAPDH was used for normalization of target genes expression. We synthesized cDNA using 200 ng of RNA and performed reverse transcription reaction with AMV Reverse Transcriptase, Oligo (dT)15 Primer and recombinant RNasin Ribonuclease Inhibitor kits (all by Promega Corporation, Wisconsin, USA). RT-PCR was performed using Sso Fast EvaGreen Supermix (Bio-Rad Laboratories, California, USA) according to manufacturer’s instructions. We used a cDNA amount corresponding to a starting RNA concentration of 5,71 ng for *DCTN* and *TFRC* for each reaction and 0,57 ng (1:10) for the others. The final concentration of each primer per reaction was 5 uM. Three technical replicates were prepared for each sample. PCR were run on the C1000 Thermal Cycler with CFX96 Real-Time System (Bio-Rad Laboratories, California, USA). PCR cycling conditions were: enzyme activation 30 sec at 95C, denaturation 5 sec at 95C, annealing 5 sec at 56C and extension 20 sec at 60C (40 cycles). Results were analyzed by CFX Manager software (Bio-Rad Laboratories, California, USA) and relative expressions were calculated with DDCt method. For each gene, the Pearson correlation coefficient was calculated between the log2-transformed expression values as measured by microarray and the negative of the Ct obtained by RT-qPCR analysis.

To verify the protein levels of the selected genes, immunohistochemical analysis were performed in the patient motor nerves and SOD1^G93A^ mice model. Transgenic SOD1^G93A^ and WT mice were purchased from Charles Rivers Laboratories Internetional, Inc. (Calco, Italy), maintained and bred at the animal house IRCCS San Raffele. Male mice were analyzed at different post-natal days: 90 (disease onset) and 120 days (end-stage disease). Animals were sacrified by exposure to carbon dioxide. All procedures were approved by the Animal Experimental Committee of the San Raffaele Scientific Institute, Milan, Italy. Procedures were carried out in accordance with the IRCCS San Raffaele of Milan Animal Care and Use Guidelines.

NOP14, PRAP1, DYSPL4 and LMNA were selected because their antibodies (Ab) were available. First, all Abs were tested to find the optimal staining conditions. For the current study the best staining conditions were: polyclonal Ab to PRAP1 (Sigma, dilution 1:250), polyclonal Ab to DPYSL4 (Sigma, dilution 1:100) and polyclonal Ab to LMNA (Sigma, dilution 1:100) on paraffin embedded human nerves by immunohistochemestry; instead ployclonal Ab to NOP14 (Sigma, dilution 1:500) worked well on mouse cryosections using immunofluorescence staining. Double staining was performed with antibodies recognizing the following antigens: Ab anti Myelin Basic Protein (MBP) to recognize myelinated nerve fibers and Ab anti Neurofilament (NF) to mark axons.

The immunohistochemistry procedure was performed with NovoCastra Polymer Detection System and Ultravision LP detection system (Thermo Scientific) on paraffin sections from patients with ALS and MN used in the present study. Sections were de-paraffinize in xylene for 20–30 minuts, then rehydrated through graded alcohols. Slides were washed in running tap water for 10 minutes. Endogenous peroxidise were neutralized using Peroxidase Block for 5 minutes; subsequently slides were incubated with Protein Block for 5 minutes, washed in TBS for 2 × 5 minutes and incubated with primary antibody all night long at room temperature. Double staining was performed by consecutively applying on the same section antibodies raised in different species. Next day sections were washed in TBS for 2 × 5 minutes and then incubated with Post Primary Block for 30 minutes; after this passage NovoLink Polymer was added for 30 minutes. Peroxidase activity with DAB working solution was developed for 5 minutes. Slides were rinsed in water and counterstained with hematoxylin. Sections were then dehydrated, and mounted with Micromount Mounting Medium.

Double immunofluorescence on SOD1^G93A^ nerves was performed using Ab NOP14 and Ab NF. Sections were permeabilized for 2 min with acetone. Sections were incubated at 4C overnight with primary antibody, washed twice with PBS for 10 min, and incubated for 30 min with FITC-conjugated or TRITC-conjugated secondary antibodies, diluted 1:150 in PBS. Controls included omission of primary antibodies on parallel sections. For nerve analysis, digitalized images of fiber cross sections were obtained from sural nerve with a digital camera (Leica DFC300F) using a 40X objective of light or fluorescence microscope (Olympus BX51). All slides were interpreted by experienced neuropathologists (F.C. and A.Q.); at the time of examination, the pathologists were blinded to all clinical and pathological data.

To further establish the Schwann cells origin of the mRNA, the presence/absence of the up- and down-regulated DEG in normal and disease peripheral nerve was also assessed by comparing our data with previous gene profiling studies on wild-type and neuropathic mouse nerves (GEO accession number GSEE40610)^10^.

## Additional Information

**How to cite this article**: Riva, N. *et al*. Unraveling gene expression profiles in peripheral motor nerve from amyotrophic lateral sclerosis patients: insights into pathogenesis. *Sci. Rep.* **6**, 39297; doi: 10.1038/srep39297 (2016).

**Publisher's note:** Springer Nature remains neutral with regard to jurisdictional claims in published maps and institutional affiliations.

## Supplementary Material

Supplementary Information

## Figures and Tables

**Table 1:** Differentially expressed genes (DEGs): Up-regulated genes.

Gene ID	Gene Symbol	Name	Chromosomal location	Fold-change	P-value	Adjusted P-value	Mouse SN microarray	Neuropathic Mouse SN microarray
ILMN_1815556	*PRAP1*	proline-rich acidic protein 1	10q26.3f	4,5733	2,52E-006	9,05E-004	N.D.	N.D.
ILMN_1792356	*DPYSL4*	dihydropyrimidinase-like 4	10q26.3e	4,0085	2,01E-005	2,29E-003	Marginal	Unchanged
ILMN_1705397	*PDK2*	pyruvate dehydrogenase kinase, isozyme 2	17q21.33a	3,8222	1,51E-005	2,02E-003	Expressed	Unchanged
ILMN_2318568	*HCFC1R1*	host cell factor C1 regulator 1 (XPO1 dependent), transcript variant 3	16p13.3d	3,5308	1,28E-006	7,38E-004	High-expressed	Unchanged
ILMN_1653927	*SNORD83A*	small nucleolar RNA, C/D box 83A, small nucleolar RNA.	22q13.1d	3,3427	1,71E-005	2,15E-003	A.	A-
ILMN_1652147	*MRPL43*	mitochondrial ribosomal protein L43	10q24.31a	3,2241	1,05E-005	1,66E-003	High-expressed	Unchanged
ILMN_1661708	*LGALS7*	lectin, galactoside-binding, soluble, 7 (galectin 7)	19q13.2a	3,2097	8,71E-005	4,38E-003	High-expressed	Unchanged
ILMN_1702009	*SV2A*	synaptic vesicle glycoprotein 2A	1q21.2a	3,0984	1,24E-004	5,22E-003	N.D.	N.D.
ILMN_1701875	*ZYX*	zyxin, transcript variant 1	7q34f	3,0589	1,19E-004	5,10E-003	High-expressed	Unchanged
ILMN_1800843	*SCAMP4*	secretory carrier membrane protein 4	19p13.3h	3,0321	1,20E-004	5,11E-003	Expressed	Unchanged
ILMN_1779177	*U2AF1L4*	U2 small nuclear RNA auxiliary factor 1-like 4, transcript variant 2	19q13.12a	3,0268	6,16E-005	3,72E-003	Expressed	Unchanged
ILMN_3236224	*SNORA74A*	small nucleolar RNA, H/ACA box 74A, small nucleolar RNA	5q31.2d	2,9864	1,27E-005	1,81E-003	A	
ILMN_2123450	*FLJ43093*	FLJ43093 protein	6p21.31a-p21.2c	2,9144	1,09E-004	4,91E-003	A	
ILMN_1779875	*THY1*	Thy-1 cell surface antigen	11q23.3f	2,8812	3,73E-004	9,75E-003	Expressed	Unchanged
ILMN_1762294	*ADAMTSL4*	ADAMTS-like 4, transcript variant 2	1q21.2b-q21.2c	2,8805	3,15E-005	2,65E-003	Expressed	Unchanged
ILMN_1666976	*PLD3*	phospholipase D family, member 3, transcript variant 1	19q13.2b	2,8677	2,21E-006	8,60E-004	Expressed	Unchanged
ILMN_1696749	*LMNA*	lamin A/C, transcript variant 2	1q22c	2,8589	1,90E-007	3,22E-004	High-expressed	Increased
ILMN_1713247	*FSCN2*	fascin homolog 2, actin-bundling protein, retinal	17q25.3f	2,8089	1,40E-004	5,61E-003	N.D.	N.D.

SN = Sciatic nerve; A. = absent in the microarray; N.D. = present in the microarray but not detected; Marginal = very low levels of expression; Expressed = present in sciatic nerve, very likely in Schwann cells; High-expressed = abundant in sciatic nerve.

**Table 2:** Differentially expressed genes (DEGs): Down-regulated genes.

Gene ID	Gene Symbol	Name	Chromosomal location	Fold-change	P-value	Adjusted P-value	Mouse SN microarray	Neuropathic Mouse SN microarray
ILMN_1690894	*HSP90B3P*	Heat shock protein 90kDa beta, member 3.	1p22.2	0.0866	8.62E-008	2.94E-004	High-expressed	Increased
ILMN_3243441	*EEF1AL7*	eukaryotic translation elongation factor 1 alpha-like 7.	4q24	0.0958	9.40E-012	2.60E-007	A.	—
ILMN_1777976	*SLC25A26*	solute carrier family 25, member 26.	3p14.1	0.1295	7.54E-006	1.39E-003	Expressed	Unchanged
ILMN_2102515	*PGAM4*	phosphoglycerate mutase family member 4.	Xq21.1	0.1765	1.88E-006	8.60E-004	N.D.	N.D.
ILMN_1739622	*PPP1R12A*	protein phosphatase 1, regulatory (inhibitor) subunit 12A.	12q21.2-q21.31	0.1921	4.02E-010	5.56E-006	Expressed	Unchanged
ILMN_1696035	*C12orf8*	chromosome 12 open reading frame 8.	12q24.13	0.2117	9.57E-008	2.94E-004	High-expressed	Unchanged
ILMN_2213558	*TMED10P*	transmembrane emp24-like trafficking protein 10.	8q24.3	0.2229	1.98E-006	8.60E-004	A.	—
ILMN_2294762	*AMY1A*	amylase, alpha 1A, transcript variant 1.	1p21.1	0.2428	1.19E-006	7.01E-004	High-expressed	Unchanged
ILMN_1726327	*AMY1B*	amylase, alpha 1B.	1p21.1	0.2705	3.83E-007	3.65E-004	High-expressed	Unchanged
ILMN_1695311	*HLA-DMA*	major histocompatibility complex, class II, DM alpha.	6p21.32	0.2872	3.08E-007	3.65E-004	A.	—
ILMN_1675331	*PEG3*	paternally expressed 3.	19q13.43	0.2957	6.91E-007	5.37E-004	High-expressed	Unchanged
ILMN_1674243	*TFRC*	transferrin receptor (p90, CD71).	3q29	0.3238	6.76E-007	5.37E-004	Marginal	Unchanged
ILMN_2300695	*IKZF3*	IKAROS family zinc finger 3, transcript variant 1.	17q12	0.3246	1.91E-006	8.60E-004	N.D.	N.D.
ILMN_3241051	*LOC644907*	hCG18290.	7p13d	0,3348	1,13E-006	7,01E-004	A.	—
ILMN_2069060	*RBM11*	RNA binding motif protein 11.	21q11.2c	0,337	6,80E-006	1,35E-003	N.D.	N.D.
ILMN_2367239	*RCAN1*	regulator of calcineurin 1, transcript variant 2.	21q22.12a	0,3494	4,04E-005	2,99E-003	Expressed	Unchanged
ILMN_1776515	*MPPE1*	metallophosphoesterase 1.	18p11.21e	0,3498	1,55E-006	7,96E-004	A.	—
ILMN_1670841	*CPNE1*	copine I, transcript variant 8.	20q11.22b	0,3564	1,58E-006	7,96E-004	Expressed	Unchanged

SN = Sciatic nerve; A = absent in the microarray; N.D. = present in the microarray but not detected; Marginal = very low levels of expression; Expressed = present in sciatic nerve, very likely in Schwann cells; High-expressed = abundant in sciatic nerve.

**Table 3:** Small Nucleolar RNA (SNORNAs) enriched in DEGs.

Probe ID	GeneSymbol	Chromosomal location	Fold-change	P-value	Adjusted P-value
ILMN_1653927	*SNORD83A*	22q13.1	3.3427	1.70E-005	0.002147
ILMN_3236224	*SNORA74A*	5q31.2	2.9864	1.30E-005	0.001812
ILMN_3235154	*SNORD124*	17q21.1	2.787	4.80E-005	0.003316
ILMN_3244449	*SNORD9*	14q11.2	2.7101	1.20E-005	0.001734
ILMN_3236408	*SCARNA5*	2q37.1	2.6914	1.39E-004	0.005604
ILMN_3239932	*SNORA53*	12q23.1	2.632	5.90E-005	0.003654
ILMN_3238745	*SNORA55*	1p34.2	2.6075	2.20E-005	0.002408
ILMN_3248232	*SNORA2A*	12q13.11	2.5964	8.00E-006	0.001429
ILMN_3248394	*SNORD63*	5q31.2	2.575	1.60E-005	0.002037
ILMN_3245605	*SNORA71A*	20q11.23	2.5131	2.09E-004	0.007109
ILMN_3241016	*SCARNA1*	1p35.3	2.4616	1.00E-005	0.001659
ILMN_3238670	*SNORA47*	5q14.1	2.3529	2.65E-004	0.008049
ILMN_3248811	*SNORA27*	13q12.2	2.3438	2.90E-004	0.008516
ILMN_3235264	*SNORA48*	17p13.1d	2.308	7.00E-006	0.001346
ILMN_3247303	*SNORD85*	1p35.2∞	2.1807	1.10E-004	0.004911
ILMN_3246869	*SCARNA21*	17p13.1	2.1485	1.50E-005	0.002021
ILMN_3239574	*SNORD3A*	17p11.2	2.0318	8.00E-005	0.004301
ILMN_3243908	*SNORA43*	9q34.3	2.0222	8.50E-005	0.004352
ILMN_3239162	*SNORA31*	13q14.12	1.9989	4.00E-005	0.00299
ILMN_2085525	*SNORA32*	11q21	1.986	3.10E-005	0.002646
ILMN_3245672	*SNORD84*	6p21.33	1.9767	3.69E-004	0.009719
ILMN_3249538	*SNORA2B*	12q13.11	1.9743	9.80E-005	0.004666
ILMN_3240781	*SNORD17*	20p11.23	1.9585	9.70E-005	0.004624
ILMN_3242825	*SNORA50*	16q21	1.9111	2.96E-004	0.008589
ILMN_3241139	*SNORD57*	20p13	1.863	7.00E-006	0.001389
ILMN_3240150	*SNORA75*	2q37.1	1.8398	2.10E-005	0.00229
ILMN_3248874	*SNORD8*	14q11.2	1.8007	2.63E-004	0.008012
ILMN_3242448	*SNORA11D*	Xp11.22	1.7561	1.82E-004	0.006503
ILMN_1689616	*SNORA66*	1p22.1	1.714	1.30E-005	0.001853
ILMN_3243921	*SNORA11E*	Xp11.22	1.7047	1.52E-004	0.00584
ILMN_3237044	*SNORD36B*	9q34.2	1.6336	3.04E-004	0.008681

**Table 4:** Pathway analysis.

	Term	ID	Statistics a	Annotated DEGs
**A Enriched Gene Ontology Terms**				
Terms enriched with up-regulated genes	Response to unfolded protein	GO:0006986	C = 127; O = 13; E = 3.21; R = 4.05; rawP = 1.96e-05; adjP = 0.0080	*SRPRB, CCND1, CALR, CUL7, HSPA4, TLN1, TSPYL2, ERO1L, BAX, CREB3L1, HSPA7, LMNA, PREB*
	Endoplasmic reticulum unfolded protein response	GO:0030968	C = 83; O = 10; E = 2.10; R = 4.76; rawP = 4.44e-05; adjP = 0.0121	*SRPRB, CCND1, CALR, CUL7, TLN1, TSPYL2, ERO1L, BAX, LMNA, PREB*
	Positive regulation of nuclease activity	GO:0032075	C = 65; O = 8; E = 1.64; R = 4.86; rawP = 0.0002; adjP = 0.0306	*SRPRB, CALR, HMGB2, CUL7, TLN1, TSPYL2, LMNA, PREB*
Terms enriched with down-regulated genes	Transferase activity, transferring nitrogenous groups	GO:0016769	C = 22; O = 4; E = 0.27; R = 14.71; rawP = 0.0001; adjP = 0.0141	*CCBL2, AGXT2L2, ABAT, GATM*
**B Enriched Reactome pathways**				
	**Pathways**	**ID**	**Statistics b**	**Annotated DEGs**
Pathways enriched with up-regulated genes	Unfolded Protein Response	381119	Gene Ratio = 8/143; BgRatio = 65/6196; pvalue = 1.09E-004; adjP = 2.85E-003	*LMNA, PREB, ATF4P3, CUL7, SRPRB, TSPYL2, TLN1, CALR*
	Activation of Chaperone Genes by XBP1(S)	381038	Gene Ratio = 6/143; BgRatio = 46/6196; pvalue = 5.95E-004; adjP = 7.28E-003	*LMNA, PREB, CUL7, SRPRB, TSPYL2, TLN1*
	Activation of Chaperones by IRE1alpha	381070	Gene Ratio = 6/143; BgRatio = 49/6196; pvalue = 8.40E-004;adjP = 7.28E-003	*LMNA, PREB, CUL7, SRPRB, TSPYL2, TLN1*
	Metabolism of proteins	392499	Gene Ratio = 25/143; BgRatio = 573/6196; pvalue = 1.26E-003; adjP = 8.21E-003	*LMNA, PREB, ATF4P3, FBXW9, PIGQ, EIF5B, PRKCSH, TOMM40, CUL7, SRPRB, EIF5A, RPL10A, TSPYL2, USP11, TUBB2A, SLC30A5, TLN1, ARSD, PIGW, INS-IGF2, CALR, IDH3G, ERO1L, F7, SPCS2*

a and b: Statistics according to WebGestalt. C: n. of genes belonging to the term in GO database; O: n. of observed genes; E: n. of expected genes; R: ratio O/E; rawP: raw p-value for hypergeometric test in WebGestalt; adjP: p-value for hypergeometric test adjusted for Benjamini-Hochberg correction. GeneRatio: ratio between the number of DEGs in the pathway and the number of DEGs. BgRatio: ratio between the number of genes in the pathway and the total examined background of genes.

**Table 5:** Over-represented Gene Ontology Terms in detected subnetworks.

	Term	ID	Statistics	Annotated DEGs
**SUBNETWORK-1**	adenylate cyclase-inhibiting G-protein coupled receptor signaling pathway (BP)	GO:0007193	C = 41; O = 3; E = 0.03; R = 89.01; rawP = 4.33e-06; adjP = 5.11e-05	*ADCY3, OPRL1, ADORA1*
	negative regulation of transmission of nerve impulse (BP)	GO:0051970	C = 37; O = 3; E = 0.03; R = 98.63; rawP = 3.16e-06; adjP = 5.11e-05	*HCRT, AVP, ADORA1*
	G-protein coupled peptide receptor activity (MF)	GO:0008528	C = 101; O = 3; E = 0.08; R = 37.48; rawP = 5.91e-05; adjP = 0.0002	*CXCR5, OPRL1, CCR2*
**SUBNETWORK-2**	rRNA processing (BP)	GO:0006364	C = 101; O = 4; E = 0.04; R = 105.99; rawP = 1.52e-08; adjP = 2.78e-07	*UTP23, NOP14, RRP8, NOP56*
	nucleolus (CC)	GO:0005730	C = 1479; O = 6; E = 0.67; R = 8.89; rawP = 5.12e-06; adjP = 0.0001	*UTP23, NOP14, RRP8, DDX52, NAT10, NOP56*
**SUBNETWORK-3**	microtubule cytoskeleton organization (BP)	GO:0000226	C = 263; O = 3; E = 0.12; R = 25.44; rawP = 0.0001; adjP = 0.0052	*KIF2A, CLASP2, TUBGCP2*
	microtubule organizing center (CC)	GO:0005815	C = 462; O = 4; E = 0.18; R = 22.13; rawP = 1.16e-05; adjP = 0.0002	*KIF2A, ARL6, CLASP2, TUBGCP2*
	purine ribonucleotide binding (MF)	GO:0032555	C = 1750; O = 3; E = 0.76; R = 3.97; rawP = 0.0298; adjP = 0.0684	*KIF2A, SRPRB, ALR6*

Statistics according to WebGestalt. C: n. of genes belonging to the term in GO database; O: n. of observed genes; E: n. of expected genes; R: ratio O/E; rawP: raw p-value for hypergeometric test in WebGestalt; adjP: p-value for hypergeometric test adjusted for Benjamini-Hochberg correction. BP: Biological Process, MF: Molecular Function, CC Cellular Component.
