# Supplementary material for: Unraveling gene expression profiles in peripheral motor nerve from amyotrophic lateral sclerosis patients: insights into pathogenesis
Source: Sci Rep. 2016 Dec 16;6:39297. doi: 10.1038/srep39297 (PMC5159906; doi:10.1038/srep39297)
Supplement: Supplementary Information [file srep39297-s1.pdf]

Unraveling gene expression profiles in peripheral motor nerve from amyotrophic lateral sclerosis patients: insights into pathogenesis

Nilo Riva[1,*], Ferdinando Clarelli[2,*], Teuta Domi[1], Federica Cerri[1], Francesca Gallia[3], Amelia Trimarco[4], Paola Brambilla[2], Christian Lunetta[5], Alberto Lazzerini[6], Giuseppe Lauria[7], Carla Taveggia[4], Sandro Iannaccone[8], Eduardo Nobile-Orazio[3], Giancarlo Comi[1,9], Maurizio D'Antonio[10], Filippo Martinelli-Boneschi[2,a], Angelo Quattrini[1,a].

[1]Experimental Neuropathology Unit, Institute of Experimental Neurology, Division of Neuroscience, San Raffaele Scientific Institute, Milan, Italy; [2]Laboratory of Genetics of Complex Neurological Disorders, Institute of Experimental Neurology, Division of Neuroscience, San Raffaele Scientific Institute, Milan, Italy; [3]2Neurology, IRCCS Istituto Clinico Humanitas, Milano University, Milan, Italy; [4]Axo-glia interactions Unit, Institute of Experimental Neurology, Division of Neuroscience, San Raffaele Scientific Institute, Milan, Italy; [5]NEuroMuscular Omnicentre (NEMO), Niguarda Ca Granda Hospital, Milan, Italy; [6]Hand Surgery and Microsurgery Unit, IRCCS Humanitas Clinical Institute, Milan, Italy; [7]3rd Neurology Unit, IRCCS Foundation "Carlo Besta" Neurological Institute, Milan, Italy; [8]Department of Clinical Neurosciences, San Raffaele Scientific Institute, Milan, Italy; [9]Universita` Vita e Salute San Raffaele, Milan, Italy; [10]Division of Genetics and Cell Biology, San Raffaele Scientific Institute, Milan, Italy.

* These authors contributed equally to this work
a These authors share senior authorship.

Correspondence to: Dr. Angelo Quattrini, Experimental Neuropathology Research Unit, Institute of Experimental Neurology INSPE, Division of Neuroscience, San Raffaele Scientific Institute, Via Olgettina, 60, 20132 Milan, Italy. Telephone number: +39-02-26435094; Fax number: +39-02-26435094; E-mail address: quattrini.angelo@hsr.it

List of Supplementary Information

Supplementary Table S1. Study patients characteristics.

Supplementary Table S2. Genes Tested by Real-time RT-PCR.

Supplementary Table S3. Relative gene expression by Real-time RT-PCR.

Supplementary Figure S1. Principal Component score plot.

Supplementary Figure S2. Validation by Real-time RT-PCR

Supplementary Figure S3. Validation of Array results by Real-time RT-PCR

Supplementary Figure S4. Active sub-network-1 and over-represented specific GO terms

Supplementary Figure S5. Active sub-network-2 and over-represented specific GO terms

Supplementary Table S1. Study patients characteristics

ID	Age	Sex	Pathologic Diagnosis	Clinical Diagnosis	Disease duration	Onset	ODSS	ALS-FRS-R	Treatment
MN-6	69.0	M	Definite MN	MN, possible disimmune aetiology	11	LL, as	2		IVIG
MN-1	68.0	M	Probable MN	Idiopatic MN	30	LL, sym	3		CSS
MN-7	76.0	M	Probable MN	MN, possible disimmune aetiology	12	LL+UL	3		CSS
MN-3	73.0	F	Probable MN	Idiopatic MN	120	LL, sym	3		-
MN-2	39.0	F	Definite MN (dem)	MN, disimmune neuropathy	108	LL, as	2		IVIG, RTX
MN-4	47.0	F	Definite MN (dem)	Idiopatic MN	56	LL, sym	2		AZA
MN-8	32.0	F	Definite MN (dem, inf)	MN, disimmune neuropathy	7	LL, sym	6		IVIG, RTX, AZA
ALS-7	44.0	M	Probable MND	ALS	49	LL, as		41	Riluzole
ALS-2	44.0	M	Probable MND	ALS	12	LL, as		44	Riluzole
ALS-8	50.0	M	Probable MND	ALS	12	LL, as		44	Riluzole
ALS-10	66.0	M	Probable MND	ALS	25	LL, sym		34	Riluzole
ALS-1	57.0	M	Probable MND	ALS	36	LL, as		39	Riluzole
ALS-9	69.0	F	Probable MND	ALS	10	LL, as		33	Riluzole
ALS-3	67.0	M	Probable MND	ALS	10	UL, as		35	Riluzole
ALS-4	63.0	M	Probable MND	ALS	12	LL, sym		38	Riluzole

Pathologic diagnosis was made according to diagnostic criteria previously established by our group (Riva et al., 2011). Briefly, a diagnosis is supported by specific histopathological signs of neuropathy, the presence of clusters of regeneration or demyelinating features (more specific features of MN: Dem: signs of demyelination; inf: signs of inflammation); a diagnosis of MND is supported by signs of axonal degeneration and the absence of clusters of regeneration. Age: years at time of biopsy; Disease duration: at time of biopsy, months; ODSS: overall disability sum score; ALS-FRS: amyotrophic lateral sclerosis functional rating score; MN: Motor Neuropathy; ALS: amyotrophic lateral sclerosis; LL. Lower limbs; UL: upper limbs; as: asymmetrical; sym: symmetrical. Medications: since nerve biopsy was performed for diagnostic purposes, specific medications were instituted after the procedure: IVIG: Intravenous immunoglobulin (IVIG); CSS: corticosteroids; RTX: Rituximab; AZA: Azathioprine.

Figure S1

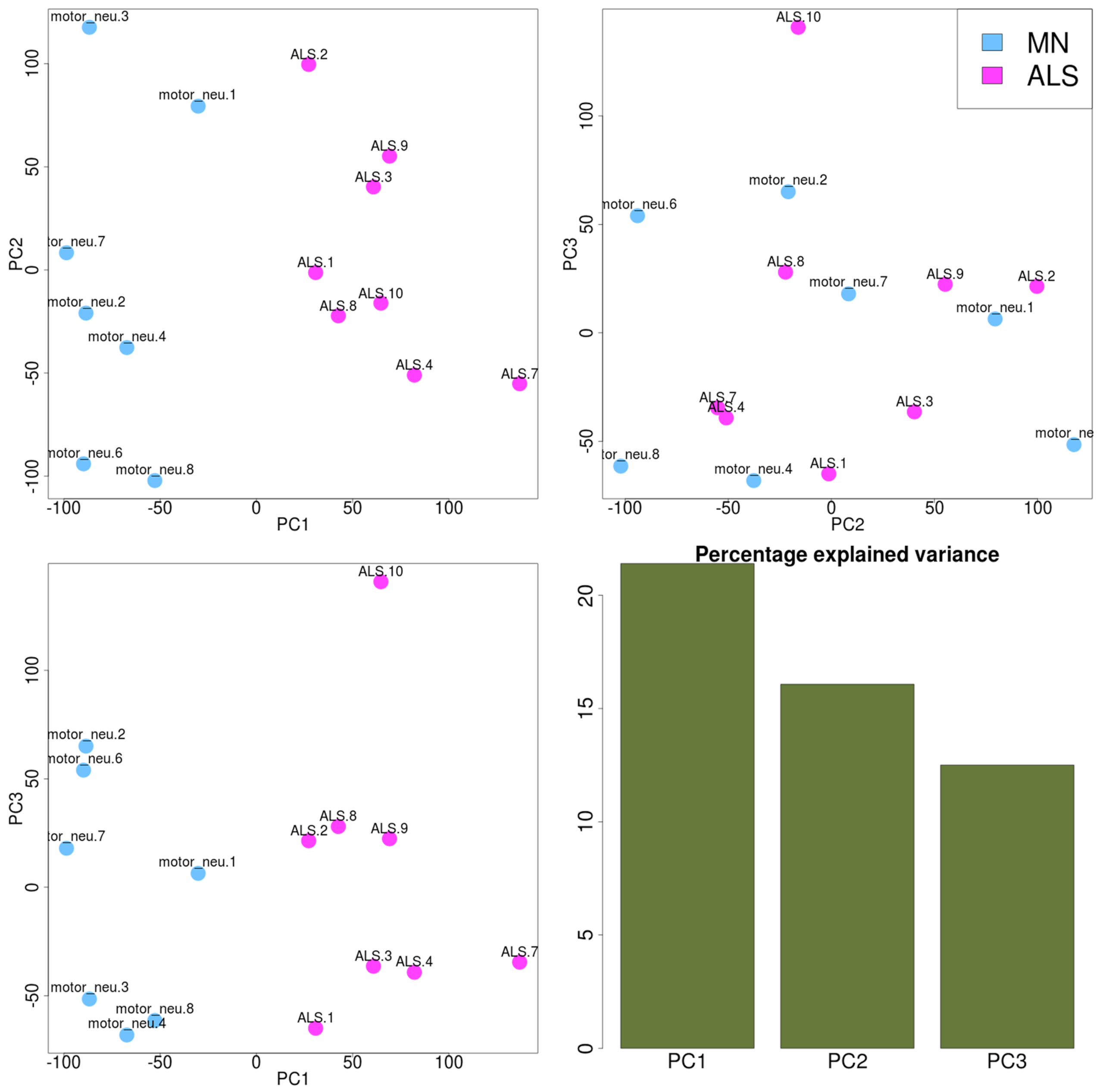

Principal Component score plot. Normalized expression values of the filtered probes for the 15 samples are projected onto the space defined by combinations of the first 3 PCs. A bar plot of explained gene expression variance is reported in the bottom-left part of the figure.

Supplementary Table S2. Genes Tested by Real-time RT-PCR

Probe ID	Symbol	Description	Correlation
ILMN_1696749	*LMNA*	Homo sapiens lamin A/C transcript variant 2	0.72
ILMN_1792356	*DPYSL4*	Homo sapiens dihydropyrimidinase-like 4	0.67
ILMN_1674243	*TFRC*	Homo sapiens transferrin receptor (p90, CD71)	0.65
ILMN_1750052	*NOP14*	Homo sapiens NOP14 nucleolar protein homolog (yeast)	0.49
ILMN_1652147	*MRPL43*	Homo sapiens mitochondrial ribosomal protein L43, nuclear gene encoding mitochondrial protein, transcript variant 2	0.10

Technical validation of the microarray data was performed on 5 selected genes (with a minimum fold-change of 2.0 in microarray) by real-time RT-PCR (3 samples/group). A Pearson correlation coefficient between the microarray results and Ct values by qRT-PCR was calculated for each gene.

Supplementary Table S3. Relative gene expression by Real-time RT-PCR.

Patients	LMNA FC	LMNA SD	NOP14 FC	NOP 14 SD	MRPL43 FC	MRPL43 SD	DPYSL4 FC	DPYSL4 SD	TFRC FC	TFRC SD
ALS2	2.095	0.092	2.085	0.091	1.839	0.181	3.451	1.770	1.037	0.065
ALS8	2.090	0.247	1.603	0.067	0.699	0.041	4.594	1.524	0.529	0.040
ALS10	1.849	0.069	1.505	0.083	1.073	0.221	1.477	0.107	0.970	0.105
MN2	1.00	0.062	1.00	0.055	1.00	0.063	1.00	0.543	1.00	0.050
MN3	0.783	0.074	1.804	0.058	1.455	0.084	1.798	0.543	2.046	0.066
MN6	1.871	0.111	1.221	0.075	0.924	0.058	1.165	0.135	1.276	0.067

For each patient, relative expression of tested genes is reported as fold change (FC) calculated by the “delta-delta Ct method”, using GAPDH as house-keeping gene and patient MN2 as reference (SD= Standard Deviation of the assay performed in triplicate).

Figure S2

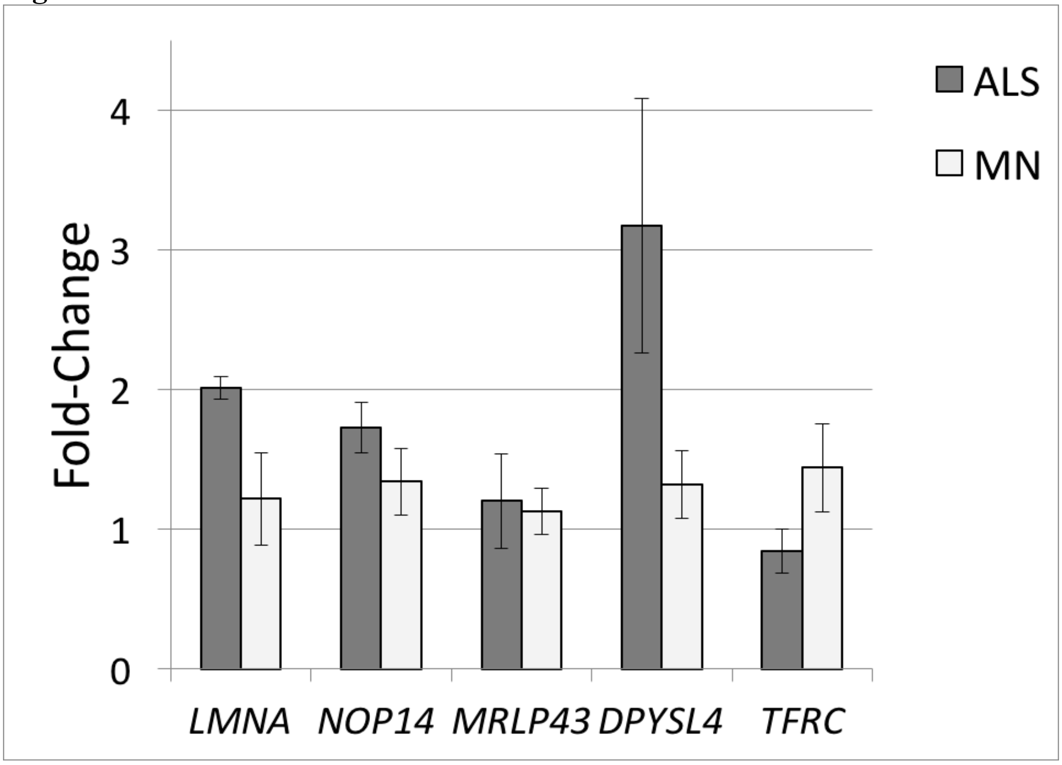

Validation by Real-time RT-PCR. Mean fold change values (Bars: SEM) from the four selected up-regulated genes (LMNA, NOP14, MRPL43, DPYSL4) and one down-regulated gene (TFRC).

Figure S3

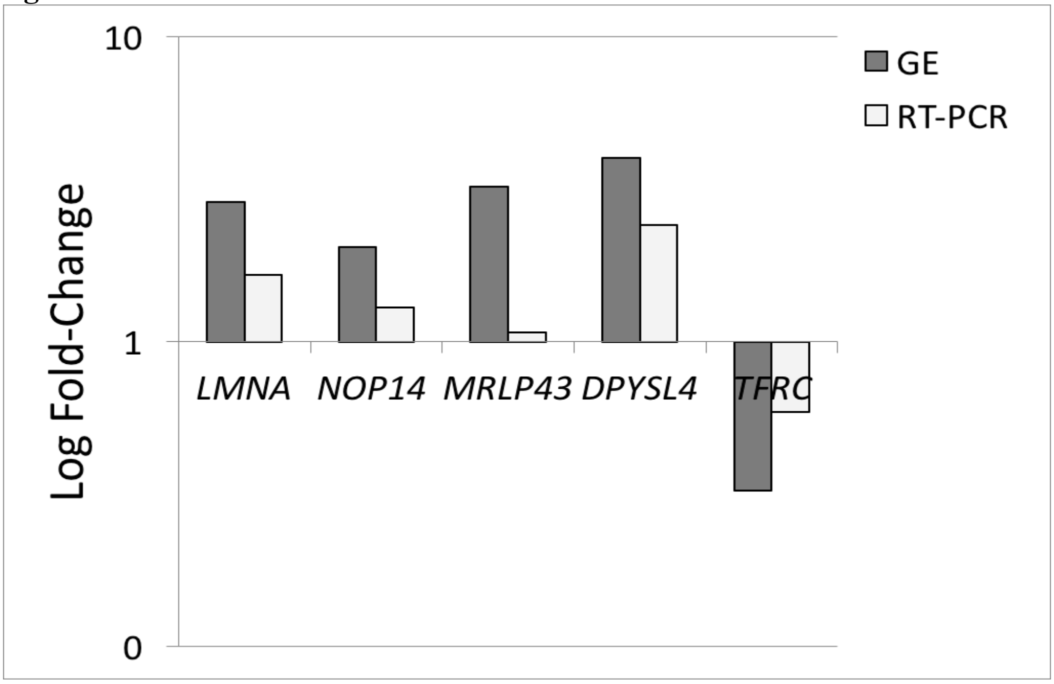

Comparision of log 2 fold changes expression values (ALS and MN) for both gene expression array and qRT-PCR.

Figure S4

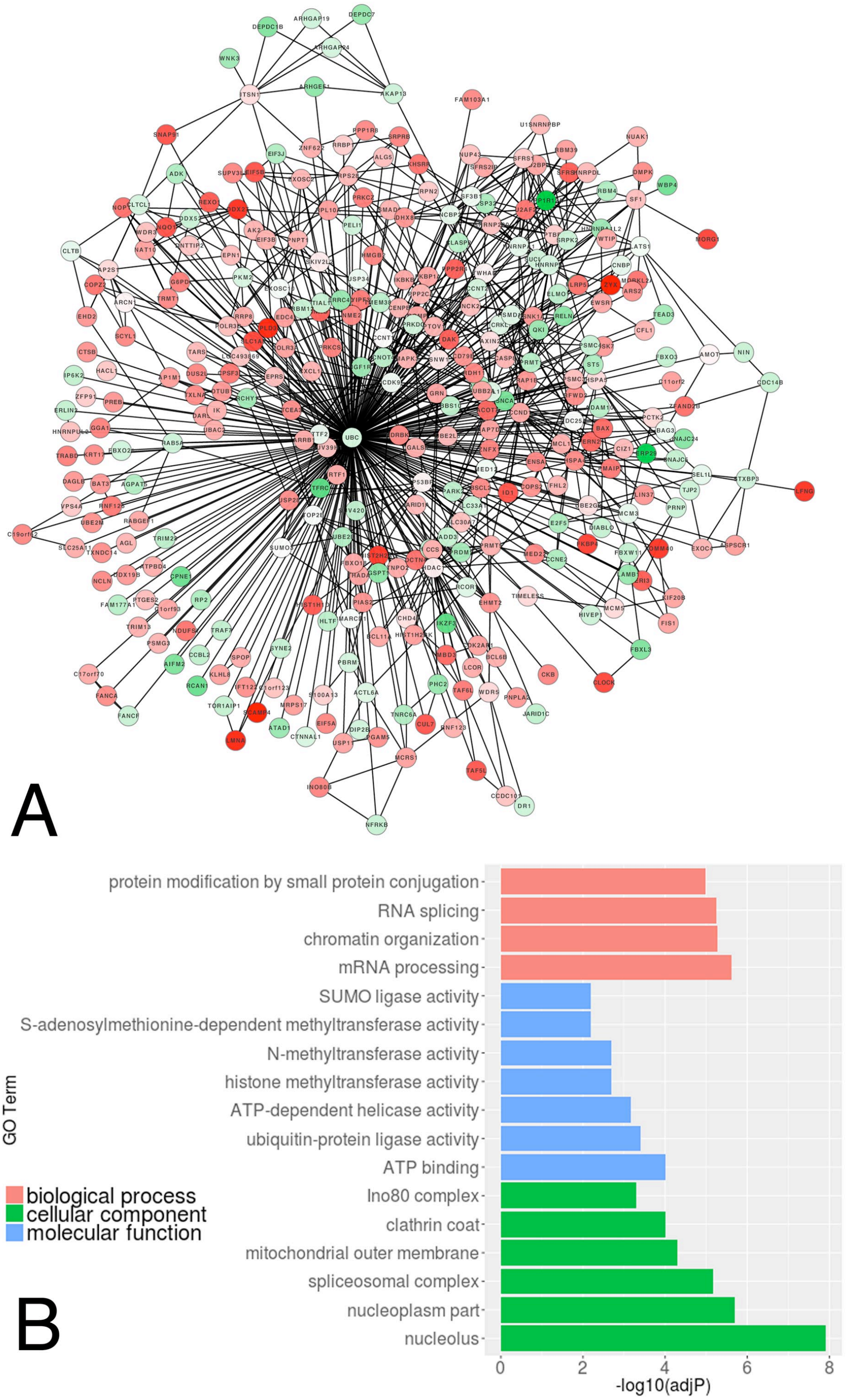

Active sub-network 1 (A) and over-represented specific GO terms (B). (A) Nodes are color-coded according to values of log2 (fold-change), with red and green nodes representing up-regulated and down-regulated genes respectively. The bar plot reporting the most specific enriched terms (B), in Gene Ontology hierarchy for the module, for each of the three ontologies. Y-axis: GO Terms; X-axis: -log10(adjP), where adjP is the enrichment p-value from hypergeometric test adjusted with Benjamini-Hochberg procedure.

Figure S5

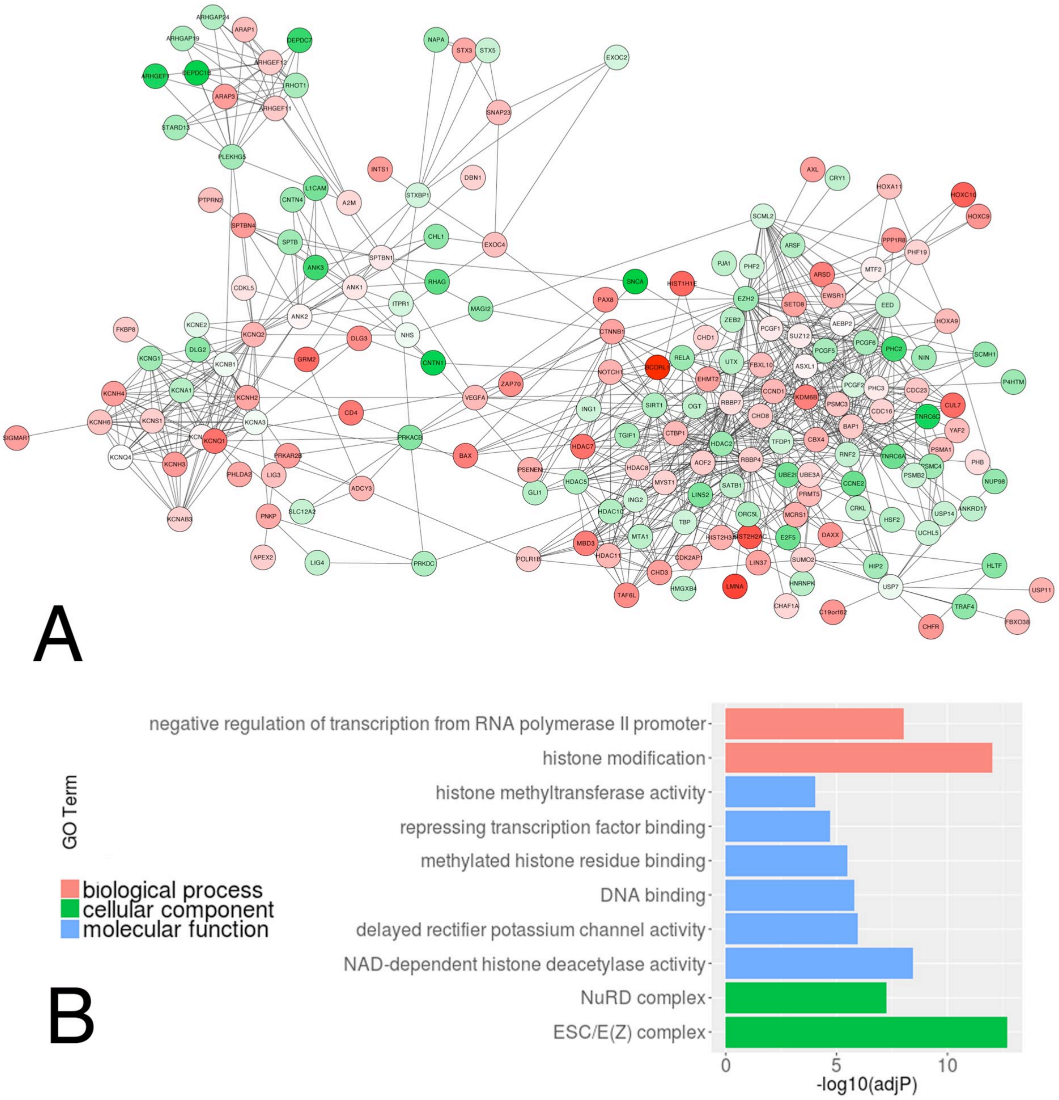

Active sub-network 2 (A) and over-represented specific GO terms (B). (A) Nodes are color-coded according to values of log2 (fold-change), with red and green nodes representing up-regulated and down-regulated genes respectively. The bar plot reporting the most specific enriched terms (B), in Gene Ontology hierarchy for the module, for each of the three ontologies. Y-axis: GO Terms; X-axis: -log10(adjP), where adjP is the enrichment p-value from hypergeometric test adjusted with Benjamini-Hochberg procedure.
